# Preliminary evaluation of the MLAA algorithm with the Philips Ingenuity PET/MR

**DOI:** 10.1186/2197-7364-1-S1-A33

**Published:** 2014-07-29

**Authors:** Alexandr Lougovski, Georg Schramm, Jens Maus, Frank Hofheinz, Jörg van den Ho

**Affiliations:** PET Center, Institute of Radiopharmaceutical Cancer Research, Helmholtz-Zentrum Dresden-Rossendorf, Bautzner Landstrae 400, 01328 Dresden, Germany; Department of Nuclear Medicine, University Hospital Carl Gustav Carus, Technische Uni-versität Dresden, 01307 Dresden, Germany

Combined PET/MR is a promising tool for simultaneous investigation of soft tissue morphology and function. However, contrary to CT, MR images do not provide information on photon attenuation in tissue. In the currently available systems issue is solved by synthesizing attenuation maps from MR images using segmentation algorithms. This approach has been shown to provide reason-able results in most cases. However, sporadically occurring segmentation errors can cause serious problems. Recently, algorithms for simultaneous estimation of attenuation and tracer distribution (MLAA) have been introduced. So far, validity of MLAA has mainly been demonstrated in simulated data. We have integrated the MLAA algorithm [2] into the THOR reconstruction [1]. An evaluation of MLAA was performed using both phantom and patient data acquired with the Ingenuity PET/MR.

Phantom data were acquired using a whole body phantom with three cylindrical inserts filled with different substances (plastic, air, glycerol). MLAA-estimated mu-maps of the phantom were compared to the mu-maps resulting from transmission measurements with an ECAT HR+ scanner. We also performed a first qualitative evaluation of the attenuation maps obtained in patient studies.

Evaluation of the phantom study showed good concordance between measured and estimated attenuation coefficients for all types of substances used in the phantom. Evaluation of patient data showed some substantial improvements of the MLAA attenuation maps compared to the segmented MR-based attenuation maps.

Preliminary results show that for the Philips Ingenuity PET/MR scanner the MLAA algorithm allows to obtain attenuation maps which outperform the MR based maps in several aspects. However, a more detailed analysis is still required to address the question of possible cross-talks in regions with high activity. Additionally, MLAA algorithm substantially increases computational burden leading to long processing times, which makes it currently impractical for clinical application.
